# Spatial analysis of gender variation in the prevalence of hypertension among the middle-aged and elderly population in Zhejiang Province, China

**DOI:** 10.1186/s12889-016-3121-y

**Published:** 2016-05-26

**Authors:** Li Xu, Dejian Lai, Ya Fang

**Affiliations:** Department of Statistics, School of Economics and Trade, Guangdong University of ForeignStudies, Guangzhou, 510006 Peoples Republic of China; Division of Biostatistics, University of Texas Health Science Center at Houston (UTHealth), School of Public Health, Houston, Texas 77030 USA; State Key Laboratory of Molecular Vaccinology and Molecular Diagnostics, Key Laboratory of Health Technology Assessment of Fujian Province University, School of Public Health, Xiamen University, Xiamen, 361102 Peoples Republic of China

**Keywords:** Shared Component Model (SCM), Hypertension, Middle-aged and elderly, Spatial analysis, Gender variation

## Abstract

**Background:**

Previous studies have shown that there may be gender disparities in the prevalence of hypertension; however, these studies do not address the spatial information contained in the sample which may limit the analytical results. Our study extends the existing Shared Component Model (SCM) and compares its utility with a logistic regression model to evaluate the significance of spatial information for identifying risk factors for hypertension and other non-rare diseases.

**Methods:**

A total of 1267 residents aged 45 years of age and over were included in our study, of which 48.1 % were males. The overall prevalence of hypertension was 33.2 %, with females experiencing a higher prevalence than males (35.5 % vs. 30.6 %). The research variables included body mass index (BMI), Waist -to-Height Ratio (WHtR), smoking status, alcohol consumption etc. The extended SCM is employed to investigate regional gender variations in the risk of hypertension and assess the gender variation in the middle-aged and elderly populations of Zhejiang Province in eastern China and then its performance is compared with that of a traditional multiple logistic regression model.

**Results:**

Our SCM analysis determined that the spatial pattern of hypertension risk for the middle-aged and elderly populations of Zhejiang Province in eastern China is quite different for males and females. Furthermore, Waist -to-Height Ratio (WHtR) continues to be a simple and effective predictor of hypertension risk for males at the regional level.

**Conclusions:**

We believe that the extended SCM spatial model is a useful tool for identifying risk factors at the regional level.

**Electronic supplementary material:**

The online version of this article (doi:10.1186/s12889-016-3121-y) contains supplementary material, which is available to authorized users.

## Background

Hypertension is a common chronic disease and a known risk factor for coronary heart disease, stroke and chronic renal failure and places a heavy burden on medical and social resources that affect families and society [1]. According to the World Health Organization’s (WHO) Study on Global Ageing and Adult Health (SAGE) [2], the prevalence of hypertension among people over 50 years of age ranged from 32 % in India, 78 % in South Africa, and nearly 60 % in China; however, the age distribution of hypertension in the study population was nearly identical. According to the Report on Chronic Disease Risk Factor Surveillance in China (2010) [3], eastern China (36.2 %) had a higher prevalence of hypertension while Middle China (34.1 %) and Western China (28.8 %) had relatively lower prevalence.

Risk factors for hypertension include a complex combination of genetic and environmental factors, which include age; higher body mass index (BMI) (overweight or obese); a high salt diet; moderate to heavy alcohol consumption; and a family history of hypertension [4–11]. Other studies have shown that mental stress, heavy work pressure, lack of physical activity and loneliness in later life may also be risk factors for hypertension [5–7, 12–14]. Researchers have found that gender may contribute to the variation of potential risk factors for hypertension (i.e. genetics, high BMI (overweight or obesity), smoking status, alcohol consumption and sleep duration) [15–23]. However, uncertainty remains for the association between hypertension and smoking status, income, education level and sleep duration [11, 15, 24–26].

Less attention has been paid with regard to gender specific variation in the prevalence of hypertension and potential risk factors [16–22], particularly in China [23, 27]. Most researchers use gender as an independent variable in models [5, 6] which employ traditional statistical methods such as the chi-square test, logistic regression, meta-analysis or Cox proportional hazard modeling as analytic tools [6, 7, 9, 28–30]. Unfortunately these methods do not address the spatial information contained in the sample, thus limiting the analytical results. Since the pathogenesis of hypertension is complex, utilizing location may serve as a useful surrogate for investigating the mixture of potential risk factors (i.e., life style choices, genetics, as well as environmental factors) that may underlie any spatial variation in disease risk. Applying spatial information offers further insight into the potential risk factors for hypertension that in turn can inform further guidelines for hypertension prevention and control.

The Shared Component Model (SCM) was first proposed by Knorr and Best [31] for the joint spatial analysis of two diseases and extended to multiple diseases by Held et al. [32]. The basic assumption of the SCM is that many diseases may be dependent on each other in that they share the same location. Therefore the space variation of disease risk could be decomposed into shared and disease specific components. The shared and disease specific components represent the unobserved spatially varying common and disease specific effects, respectively. By decomposition, the SCM is able to identify common geographical patterns that may identify the differences in disease burden between regions. By spatially smoothing through “borrowing strength” from adjacent areas, the SCM is able to effectively avoid the problem of instability of standardized disease rates, especially for rare diseases in small areas analysis [31, 32]. Different from traditional statistical methods, the modeling procedure of the SCM utilizes the dependency, not only among diseases, but also among spatially varying variables. The SCM can better describe the epidemiologic feature of risk factors associated with disease; therefore leading to more cost effective interventions [33]. The SCM is also regarded as an effective way to conduct spatial analysis for multiple diseases [34–37]. Recently, the SCM has been applied to investigating the regional variation of gender inequalities in hospital admissions for chronic diseases in elderly people, as well as gender variation in the spatial pattern of alcohol-related mortality [38, 39].

In this paper we employ the Shared Component Model (SCM) to assess regional gender variation in the risk of hypertension in the middle-aged and elderly populations of Zhejiang Province in eastern China. Our study utilizes the SCM and compares its utility with a logistic regression model to evaluate the significance of spatial information for identifying risk factors for hypertension and other non-rare diseases.

## Methods

### Data source

We obtained data from China Health and Retirement Longitudinal Study (CHARLS, 2012) [40] conducted by the Peking University National Development Research Institute, Chinese Economic Research Center, a research project designed to collect health data from a nationally representative sample of Chinese residents 45 years of age or over to augment the needs of scientific research in the elderly Chinese population. Details of the sampling procedure are available at http://charls.ccer.edu.cn/zh-CN. The datasets for descriptive in Table 1 and logistic analysis in Table 2 can be found in the Additional file 1, while the dataset for SCM analysis in Table 3 can be found in the Additional file 2.

**Table 1:** Characteristics of subjects reporting ever being diagnosed with hypertension according to gender

	Males, % (counts)	Females, % (counts)
Age (years of age)		
45–59	20.43 (48/235)	25.41 (78/307)
60–74	34.43 (94/273)	45.06 (114/253)
75-	44.00 (44/100)	42.71 (41/96)
Smoke		
Never	33.52 (60/179)	35.36 (227/642)
Current	29.44 (126/428)	42.86 (6/14)
Alcohol consumption		
Never	29.35 (59/201)	35.00 (182/520)
Current	40.86 (38/93)	41.82 (23/55)
BMI (kg/m^2^)		
<18.5	20.00 (6/30)	29.03 (9/31)
18.5–23.9	25.81 (64/248)	24.79 (60/242)
24.0–27.9	39.50 (47/119)	48.73 (77/158)
≥28.0	44.83 (13/29)	54.55 (30/55)
WHtR		
≤0.5	18.59 (29/156)	17.65 (15/85)
>0.5	37.87 (103/272)	39.95 (163/408)

*BMI* Body mass index, *WHtR* Waist-to-height ratio; The datasets supporting this findings can be found in the Additional file 1

**Table 2:** Risk factors of hypertension by multiple logistic regressions according to gender

Variables	Males		Females	
	OR	95 % CI	OR	95 % CI
Age (years of age)				
45–59	1.00	_	1.00	_
60–74	2.67*	1.58–4.54	2.76*	1.78–4.28
75-	4.86*	2.46–9.58	2.03*	1.06–3.82
BMI (kg/m^2^)				
<18.5	0.69	0.24–1.92	1.44	0.56–3.73
18.5–23.9	1.00	_	1.00	_
24.0–27.9	1.68	0.97–2.91	2.96*	1.87–4.80
≥28.0	2.00	0.85–4.71	3.45*	1.82–6.53
WHtR				
≤0.5	1.00	_	1.00	_
>0.5	1.85*	1.03–3.34	1.84	0.89–3.78

*BMI* Body mass index, *WHtR* Waist-to-height ratio, *CI* Confidence Interval, *p-value < 0.05; The datasets supporting this findings can be found in the Additional file 1

**Table 3:** Relative risk of risk factors by SCM modeling according to gender

Parameter	Males		Females	
	Mean	95 % C.I.	Mean	95 % C.I.
RR for age	0.965	0.931–1.000	1.012	0.990–1.034
RR for obesity	0.928	0.852–1.007	1.019	0.953–1.089
RR for WHtR	1.035	1.008–1.063	1.020	0.997–1.044
_*η*_	0.495	0.021–0.975	0.496	0.022–0.973
_*σind*_	0.017	0.005–0.062	0.017	0.005–0.061
_*σspat*_	0.018	0.005–0.064	0.018	0.005–0.069
_*σstr*_	0.018 (0.005–0.064)			
_*σunstr*_	0.016 (0.005–0.056)			
_*δ*_	1.094 (0.454–2.197)			
DIC (pD)	DIC = 116.188 (pD = 8.327)			

*RR* Relative Risk, *DIC* Deviance information criterion, *WHtR* Waist-to-height ratio; The datasets supporting this findings can be found in the Additional file 2

### Study areas

**Fig. 1:**
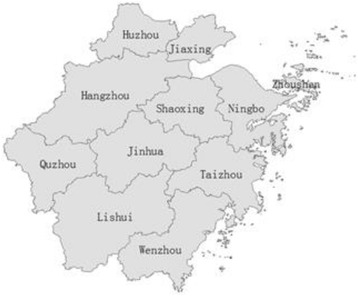
The administrative map of Zhejiang Province

Our selected study area, Zhejiang Province, is located in the south wing of the Yangtze River on the southeast coast of China, which is divided into northeast and southwest Zhejiang by geomorphological features (abbreviated as ZN and ZS, respectively). The geomorphic type of ZN is a plain and consists of six cities (Huzhou, Jiaxing, Zhoushan, Hangzhou, Ningbo, and Shaoxing), while ZS is dominated by hills, mountains and is comprised of five cities (Jinhua, Quzhou, Taizhou, Lishui, and Wenzhou) (Fig. 1. The administrative map of Zhejiang Province). The economic development of Zhejiang Province is so robust that its per capita disposable income for residents has ranked first for twenty-one consecutive years in China, with ZN significantly higher than ZS due to the different natural and geographical environment. Zhejiang Province has a higher proportion of the aging population in China, including 48.62 million inhabitants, of whom 19.4 % were 60 years of age or over in 2014 [41].

### Research variables

Study participants included individuals who were 45 years of age or over. Hypertension was self-reported [24] and defined by “have you been diagnosed with hypertension by a health professional”. Using the appropriate guidelines from the Global Burden of Disease (2000) [42], our study participants were grouped into middle-aged (45–59 years of age), young elderly (60–74 years of age) and older (75 years of age and over) age categories. Full-time education was categorized as illiterate; primary school; junior high school; high school and secondary specialized school; and college or above. Sleep duration included the average sleep time in hours at night and naptime in minutes during the past month. Smoking status was assigned as current smoking, the amount of smoking, duration of smoking, quit smoking and smoking cessation period. Alcohol consumption was measured to include current alcohol consumption, duration of drinking, quit drinking, and drinking cessation period.

In terms of overweight and obesity status, the variables included body mass index (BMI) and waist to height ratio (WHtR). The measurements of weight, height and waist circumference are almost the same as Health and Retirement Study (HRS) [43]. Based on the recommendations of the Obesity Problem Working Group of Chinese Adults in 2001 [44], BMI was grouped into four categories: <18.5 kg/m^2^ (underweight), 18.5–23.9 kg/m^2^ (normal weight), 24.0–27.9 kg/m^2^ (overweight), and >28.0 kg/m^2^ (obese). Waist-to-height ratio (WHtR) is the ratio of waist circumference to height, with 0.5 as the cut point [45, 46]. A WHtR > 0.5 represented an “apple shaped” body and a WHtR ≤ 0.5 represented a “pear shaped” body.

### Statistical methods

Univariate and multiple logistic regressions were performed in the traditional analysis. Variables with *p*-values < 0.06 were selected by univariate logistic regression to enter into the multiple logistic regression models [7] to ensure that all potential risk factors were taken into consideration. Because the risk of hypertension is closely related with life style choices, we chose age, BMI, WHtR, smoking status, alcohol consumption and sleep duration as covariates for the multivariate logistic regression models.

We employed the SCM for the spatial analysis with inference performed in a Bayesian framework using Markov chain Monte Carlo (MCMC) methods. Because the multiple logistic regression models were conducted separately for males and females, different intercept and beta were used for each gender as in the SCM.

#### Shared Component Modeling (SCM)

Standardized prevalence, defined as the ratio of observed to expected count in the region under study, is commonly used for mapping the geographic dispersion of disease prevalence. The modeling procedure for SCM can be found in Knorr and Best, Held et al., and Ibanez-Beroiz et al. [31, 32, 38]. However, the models used in these studies most often did not include covariates or potential fixed effects between different diseases (or gender). Furthermore, these investigators mainly focused on rare diseases and adopted the Poisson distribution. Because the prevalence of hypertension is quite common, a binomial distribution was more appropriate for our model settings [34]. Therefore, our study broadens the existing SCM by pinpointing fixed effects according to disease or gender specific regional covariates and extends its application to hypertension, a non-rare disease. To account for regional information, the covariates selected were self-reported smoking, alcohol consumption and obesity. Based on the data collection methods of the China Health and Retirement Longitudinal Study, our study assumed that the sample was a representative sample of the Chinese population, aged 45 years and over. Thus, the extended SCM of our study can be presented as follows:

1 $$ O\left[j,i\right] \sim bin\left(n\left[j,i\right],p\left[j,i\right]\right) $$

2 $$ \log \kern0.1em  it\left(p\left[j,i\right]\right)={\displaystyle {\alpha}_j}+{\displaystyle {\beta}_j}\ast {\displaystyle {x}_{j,i}}+ eta\left[j,i\right] $$

3 $$ eta\left[1,i\right]=\varphi \left[i\right]\ast \delta +\upsilon \left[1,i\right] $$

4 $$ eta\left[2,i\right]=\varphi \left[i\right]/\delta +\beta \left[i\right]+\upsilon \left[2,i\right] $$

5 $$ {\displaystyle {\eta}_1}=\operatorname{var}\left(\varphi \left[i\right]\ast \delta \right)/\left(\operatorname{var}\left(\varphi \left[i\right]\ast \delta \right)+\operatorname{var}\left(\upsilon \left[1,i\right]\right)\right) $$

6 $$ {\displaystyle {\eta}_2}=\operatorname{var}\left(\varphi \left[i\right]/\delta \right)/\left(\operatorname{var}\left(\varphi \left[i\right]/\delta \right)+\operatorname{var}\left(\beta \left[i\right]\right)+\operatorname{var}\left(\upsilon \left[2,i\right]\right)\right) $$

where *i* denotes region (i = 1,2,…,11), *j* represents gender with j = 1 for males and 2 for females, and *x*_*j,i*_ was the regional level independent variables, and implications for other variables included:

*O[j,i]* and *n[j,i]*: the observed counts of cases and total number of observations, respectively;

*eta[j,i]* and *ν[j,i]*: overall and gender specific spatial variation in hypertension relative risk(RR);

φ*[i]:*common spatial variation for both genders;

*β[i]:* spatial patterns of gender variation in hypertension RR adjusted for potential confounders;

*δ and 1/δ:* weights of shared component for males and females respectively with their ratio equals to *δ*^*2*^; and

*η*_*j*_: proportion variance shared for males and females, respectively;

Of note, the weighting method mentioned above set the sum of the logarithm weight equal to 0, ensuring identifiability of the model.

Because Bayesian inference was used for model fitting and requires the specification of prior distribution for model’s parameters and hyper-parameters, we referred to the work of Arul Earnest et al. [37] and incorporated the structure of our dataset.

Convolution prior was adopted for *φ[i]* and *ν[j,i]* [47], denoting they both may be decomposed into the sum of unstructured (e.g. ush, ssh) and spatial structured random effects (e.g. bind, bspat):

7 $$ \varphi \left[i\right]=ush\left[i\right]+ssh\left[i\right] $$

8 $$ \upsilon \left[j,i\right]= bind\left[j,i\right]+ bspat\left[j,i\right] $$

Then ush and bind were all assigned Normal prior with their mean zero and *α*_*0*_*[j]* respectively, while that of ssh, bspat and *β[i]* were Conditional Autoregressive (CAR) Normal prior and *vunstr, vind[j], τ*_*ssh*_*,τ*_*bspat*_ and *τ*_*β*_ are precision parameters for the corresponding prior distributions.

And the fixed effect *α[j]* and hyper-parameters *α*_*0*_*[j]* were both assumed to be a non-informative prior. The logarithmic of weights *δ* was assumed N(0.0, 0.169) [37], which means that *δ*^*2*^ is between 1/5 and 5 with 95 % probability, regardless of whichever disease is labeled 1 or 2. As suggested by Mollie [48], in the absence of prior information, random effects of unstructured and spatially structured were assumed equally important, making all prior distributions of precision parameters the same. For example, the weakly information of gamma distribution Gamma (1, 1.0E-4) (priors 1) was assumed.

The Bayesian SCM models were fitted using Markov Chain Monte Carlo (MCMC) techniques. In order to address reliability, two parallel MCMC chains with widely different starting values were run for each model with a total of 50,000 iterations, keeping every 10th, after a 50,000 iteration burn-in period for each chain. Results were based on thinned sample sizes of 10,000. Brooks–Gelman–Rubin diagnostics [49], as well as graphical checks of chains and their autocorrelations were performed to assess convergence. After convergence, the commonly used deviance information criterion (DIC) [50] was calculated to guide model selection. Finally, to assess the sensitivity of the selected model with respect to different priors for the precision parameters, other alternatives such as Gamma (1000, 5.0E-7) (priors 2) and Gamma (5.0, 5.0E-5) (priors 3) were also considered [51].

Data extraction, management and logistic regression analysis were performed in Stata (version 11, Stata Corp, College Station, USA). The SCM models were run in the free software WinBUGS version 1.4.3 with the GEOBUGS version 1.2 add on. All weight matrices were created using GeoBUGS. Maps of Zhejiang province were first produced in ArcMap version 10.1 (ESRI, USA) and then imported into GeoBUGS.

## Results

### The characteristics of participants and prevalence of hypertension

A total of 1267 residents aged 45 years of age and over were included in our study, of which 48.1 % were males. The age proportions of participants were 38.6 %, 44.8 % and 16.6 % for middle aged (45–59 years of age), young elderly (60–74 years of age) and older (75 years of age and older) respectively among males and 47.0 %, 38.5 % and 14.6 % among females. For males and females, participants whose education were junior high school or less accounted for 90.5 % and 95 %, respectively; and their average sleeping durations were approximately seven hours, with males taking much longer naps than females (40.8 min versus (vs.) 28.9 min). For males, current smoking and alcohol consumption were more prevalent than females (70.4 % vs. 2.1 %, 31.5 % vs. 9.5 %, respectively); whereas the prevalence of obesity and a WHtR >0.5 were lower than that of females (6.8 % vs. 11.3 %, 63.6 % vs. 82.8 %, respectively) (Table 1).

The overall prevalence of hypertension was 33.2 %, with females experiencing a higher prevalence than males (35.5 % vs. 30.6 %). Table 1 describes the prevalence of hypertension for participants stratified by age, smoking status, alcohol consumption, BMI and WHtR for both genders. The prevalence of hypertension among females in each group was higher than that of males, particularly for those 60–74 years of age, obese and current smoking, with the differences ranged from 9.7 to 13.5 %-points. The prevalence of hypertension for males significantly increased with age, whereas it did not for females. Current smoking and alcohol consumption increased the risk of hypertension by approximately 7 %-points for females, whereas their effects ranged from -6 to 12 %-points for males. The prevalence of hypertension among overweight males and females increased by 14 and 24 %-points respectively, with approximately 5 %-points increments for underweight females in comparison with normal weight females. The opposite effect was found for males. Compared with WHtR ≤ 0.5, those with a WHtR > 0.5 increased the prevalence of hypertension by approximately 20 %-points for both genders. Furthermore, the effect of obesity was apparent for both males and females, as prevalence of hypertension increased approximately 20 and 30 %-points in comparison with normal weight, respectively (Table 1).

### Risk factors of hypertension by logistic regression analysis

A traditional logistic regression analysis was conducted to investigate risk factors for hypertension and used for comparison with results obtained by spatial analysis model to determine which model better identifies potential risk factors for hypertension. Univariate logistic regression was performed by regressing binary hypertension status on smoking status, alcohol consumption, education level, sleeping duration and obesity. To further address interaction with potential confounders, variables with *p*-values <0.06 in the univariate analysis were chosen to enter into a forward stepwise multiple logistic regression model^7^. In order to ensure the robustness of parameter estimations, all dependent variables used in the regression model were assessed for multi-collinearity.

Table 2 presents the results of the multivariable logistic regressions models. As age increased, the risk of hypertension for middle-aged (45–59 years of age) and elderly (60–74 years of age and 75+ years of age) males and females both increased, whereas gender differences existed in effects of BMI and WHtR. Compared with the middle-aged participants, males who were elderly had an increased risk of hypertension by 1.7 times and 3.9 times respectively, whereas they were 1.8 times and 1.0 times for females. It is worth noticing that the odds ratios (OR) were roughly the same for males and females 60–74 years of age but higher for males than females among those 75+ years and older. Finally, the risk of hypertension increased by 85 % for males whose WHtR > 0.5 compared to those whose WHtR ≤ 0.5, while overweight and obesity increased the risk of hypertension in females only by two times and 2.5 times respectively. These results confirmed that WHtR and BMI continue to be of great importance for risk of hypertension in males and females (Table 2).

### Results of SCM

#### Parameters of posterior estimation

Combining with results from the multiple logistic regression models, the proportion of the elderly (aged 60 years and over), alcohol consumption, smoking status, obesity and WHtR were used as candidate predictors to assess the correlation between hypertension and age, alcohol consumption, smoking status, obesity and WHtR in middle-aged (45–59 years of age) and elderly (60+ years and older) at the regional level. Hypertension relative risk (RR) of each city for both genders was obtained by MCMC techniques. According to DIC criterion, autocorrelation and convergence of model parameters, a model that included the proportion of the elderly, obesity and those with a WHtR > 0.5 was the most appropriate (Table 3). Further, the observations and expected cases of hypertension for both genders in Zhoushan City were substituted by the average value, and the missing covariates were assumed to have a normal distribution with the mean and variance obtained by the corresponding covariates respectively.

Results of model parameters from the SCM are shown in Table 3. WHtR > 0.5 was a risk factor for hypertension in males, whereas the impact of age and obesity (especially obesity) were uncertain (with confidence intervals containing one) at the regional level. Unlike males, the impact of these factors (especially obesity) was uncertain in females. Despite this uncertainty, the posterior mean of the relative risk of age and WHtR for females were both greater than one with the lower bound close to one. These findings suggest that these factors may significantly increase the risk of hypertension in females in most regions but not in Jinhua, ZS and Quzhou, ZS. Furthermore, obesity was a key factor for both genders in Lishui, ZS and females in the southeast coastal area. Overall, there appeared to be much more uncertainty regarding the impact of obesity on both genders which may indicate that city as a basic unit may not be the most parsimonious tool. Hence, it may be necessary to conduct analysis at finer scale (e.g., at the county level) to improve the precision of these estimations in the future research.

Comparing results of the SCM with that of the multiple logistic regression analysis to determine which model better identifies potential risk factors for hypertension indicated that the risk of hypertension was significantly increased for males whose WHtR > 0.5 at both the individual and regional levels. However, the results of the SCM also showed that a WHtR > 0.5 was also a risk factor for females in most regions (except Jinhua, ZS and Quzhou, ZS) which were not identified in the multiple logistic analysis. Furthermore, the multiple logistic regression analysis demonstrated that obesity was only significant for females. However, the SCM was able to reflect areas associated with obesity visually by mapping the spatial pattern for both genders even if the posterior credible intervals contained one. Finally, the risk of hypertension decreased slightly for males who were 60 years of age and over compared with those 45–59 years of age, contrary to the multiple logistic regression models.

Table 3 further shows that the variance of spatial structured was larger than that of unstructured for both components of shared and gender specific (eg.*σ*_*str*_ > *σ*_*unstr*_), suggesting the existence of a significant spatial effect. The fraction of variance shared was about 49 % for both genders, which indicated the variation of shared random effects was slightly less than gender-specific effects (51 %). Thus gender variation for effects of unobserved covariates in hypertension was relatively important. The posterior mean of the weight of shared components *δ* being larger than one indicated that the effects of other shared risk factors, in addition to WHtR, such as smoking status, alcohol consumption, may be more important for males. It should be noted that the posterior confidence intervals of *δ* and *η* were relatively wide due to the imprecision inherent in estimating such ratio parameters [39], which may be overcome if analyzed at a finer scale (e.g. county).^1^ The posterior estimation of parameters by the SCM identified gender variations of age and obesity in hypertension at the regional level and the risk of hypertension was significantly increased for those with a WHtR > 0.5 compared with those with a WHtR ≤ 0.5, especially among males.

#### Spatial pattern of relative risk adjusted and unadjusted for WHtR and age

**Fig. 2:**
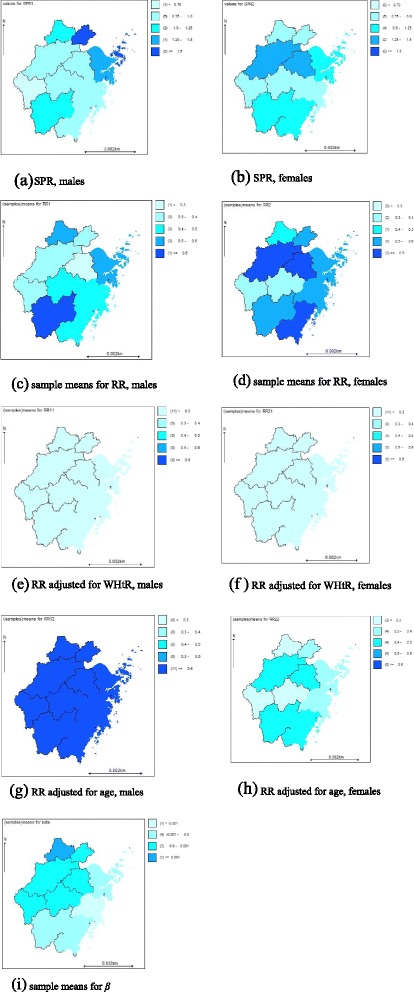
**a** - **i** denotes the SPR, the RR, the posterior mean of RR adjusted for WHtR and age and the posterior mean of β of Zhejiang Province in 2012, by gender

In order to investigate the spatial distribution of hypertension and related risk factors in the middle-aged and elderly populations, we mapped the standardized prevalence ratio (SPR), the RR, and posterior mean of gender variations of hypertension by SCM modeling on the GEOBUG map of Zhejiang Province (Fig. 2). Figure 2 (a)- (i) displays the SPR, the RR, posterior mean of RR adjusted for WHtR and age and posterior mean of *β* of Zhejiang Province in 2012 by gender. The spatial distribution of the RR adjusted for obesity can be found in Figure S2 in the Additional file 3.

Figure 2 (a) and (b) show that the spatial distribution of the SPR for males and females in ZN was larger than that of ZS, although there were slight differences. The largest values occurred in Jiaxing, ZN and Ningbo, ZN for males and Hangzhou, ZN and Shaoxing, ZN for females, whereas the lowest value was in Quzhou, ZS for males. It is well known that the SPR is the maximum likelihood estimator (MLE) of RR [35], which is usually unbiased. However, it may be unstable for areas with small populations as the small changes in the denominator may bring about greater variations in estimates. Therefore, resulting maps may be misleading [32]. To avoid this problem, we assessed the cities with the highest SPR. According to our findings, the expected cases in Jiaxing, ZN were the lowest, implying that the extreme value of SPR for males in this region was most likely due to the occasional changes in the denominators. However, the extreme effects of SPR were smoothed out after spatially smoothing by the SCM (Fig. 2 (c)). Furthermore, the RR for hypertension for females in most areas was higher than those for males after spatially smoothing, and the former showed the spatial pattern of coastal to inland and the previous pattern of SPR for the latter was no longer obvious (Fig. 2 (c) and (d)). A potential reason for the relatively high RR in some regions may be that their corresponding WHtR were larger which in shown in Table 3 and Fig. 2 (e) and (f). By the same reasoning, obesity was also a risk factor for hypertension for both genders in Lishui, ZS and females in the southeast coastal regions (Figure S2 (a) and (b) in the Additional file 3).

Figure 2 (g) and (h) show that in most areas (except Jinhua, ZS and Quzhou, ZS), the RR for hypertension for females was significantly decreased after adjustment for age whereas it was opposite for males, thus implying that 60 years of age or older was a risk factor for hypertension for females yet protective for males to some extent. Finally, Fig. 2 (i) indicates that the RR for hypertension in the southeast coastal regions for males was a bit higher than females, with the opposite trend in other regions, though not obvious. These findings suggest that there were other risk factors resulting in the gender variation of the RR after adjustment for age and obesity, consistent with the implication of *η* in Table 3.

### Convergence and sensitivity analysis of SCM modeling

**Fig. 3:**
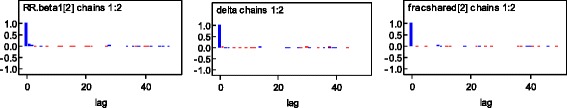
Autocorrelation of RR for WHtR, 1*/δ* and *η*

**Fig. 4:**
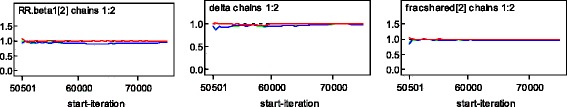
Convergence of RR for WHtR, 1*/δ* and *η*

To ensure the reliability of the estimations, convergence of the selected variables was diagnosed and the sensitivity of the selected model to the choice of priors for the precision parameters was also assessed. Figures 3 and 4 briefly describe the autocorrelation and convergence of the regression coefficients of the RR for WHtR, 1*/δ* and *η* for females (results for males were similar and can be found in Figure S3 in the Additional file 4), which showed that there was no obvious autocorrelations and convergence was achieved. The sensitivity analysis confirmed that the posterior estimates and the resulting DIC scores were both robust in regard to the moderate changes in the prior distributions, thus their appropriateness in the proposed model (Table 4).

**Table 4:** Sensitivity analysis with respect to different priors for the precision parameters

Parameter	Priors 1	Priors 2	Priors 3
	Mean (95 % CI)	Mean (95 % CI)	Mean (95 % CI)
RR for age			
Males	0.965 (0.931–1.000)	0.966 (0.931–1.001)	0.966 (0.932–1.002)
Females	1.012 (0.990–1.034)	1.012 (0.991–1.034)	1.012 (0.991–1.034)
RR for obesity			
Males	0.928 (0.852–1.007)	0.930 (0.854–1.008)	0.931 (0.856–1.010)
Females	1.019 (0.953–1.089)	1.018 (0.955–1.085)	1.019 (0.954–1.091)
RR for WHtR			
Males	1.035 (1.008–1.063)	1.034 (1.007–1.061)	1.034 (1.005–1.062)
Females	1.020 (0.997–1.044)	1.021 (0.999–1.044)	1.020 (0.997–1.043)
*η*			
Males	0.495 (0.021–0.975)	0.506 (0.109–0.897)	0.502 (0.088–0.914)
Females	0.496 (0.022–0.973)	0.498 (0.103–0.896)	0.502 (0.088–0.910)
_*δ*_	1.094 (0.454–2.197)	1.099 (0.447–2.231)	1.090 (0.448–2.247)
DIC(pD)	DIC = 116.188 (pD = 8.327)	DIC = 116.471 (pD = 8.233)	DIC = 116.196 (pD = 8.100)

*RR* Relative Risk, *DIC* Deviance information criterion, *WHtR* Waist-to-height ratio

**Fig. 5:**
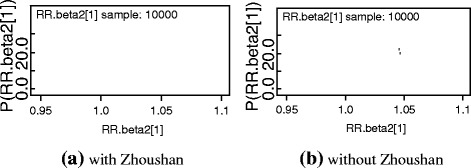
**a** and **b**, Kernel density of RR for WHtR for males. The sampling value and frequency of RR for WHtR are expressed in horizontal and vertical axis respectively. Two parallel MCMC chains were run for each model with samples size of 10,000

In order to ensure the propriety of normal assumption for Zhoushan City, ZN, a second analysis was conducted by excluding this region. We found that the distribution of the RR for WHtR were basically identical in both cases, though it was more smoothly in the second analysis (Fig. 5. Kernel density of RR for WHtR for males, results for females can be found in Figure S5 in the Additional file 5).

## Discussion

With the acceleration of an aging population, the high prevalence of hypertension in the middle-aged and elderly will not only affect one’s normal life but may also cause heavier disease burdens, at the individual, family, and global levels. Previous studies have shown that there may be gender disparities in the prevalence of hypertension; however, certain limitations in these studies may have existed in that they were most often performed using the traditional statistical methods (e.g. logistic regression) which often does not take spatial information into consideration [6, 7, 9, 28, 30]. However, the spatial aspect of location can serve as a surrogate for addressing the mixture of life style choices, genetic factors and environmental factors that often underlie the spatial variation in disease risk.

Considering the availability of data and growth of China aging population, our study employed the SCM to ascertain regional gender variations in hypertension risk for the middle-aged and elderly population in Zhejiang Province, China. Our paper focuses on a non-rare disease but broadens the existing SCM by pinpointing fixed effects according to disease or gender specific regional covariates. As a result, the SCM can be used to quantify disease or gender specific fixed effects and applied to non-rare diseases (e.g., hypertension) for identifying risk factors at the regional level.

As early as 1947, a French doctor declared that people who were “apple shaped” (WHtR > 0.5) suffered from higher risk of cardiovascular diseases compared to “pear shaped” (WHtR ≤ 0.5), as too much weight was carried in the abdomen for “apple shaped”, yet the lower for those who were “pear shaped”. The logistic regression model in our study found the same correlation in middle-aged and elderly males only, whereas our SCM analysis demonstrated this relationship for both males and females, especially in males. Moreover, the results of the SCM indicated that obesity mainly increased the risk of hypertension for both genders in ZS and females in the southeast coastal regions. Consistent with previous studies [45, 46, 52], WHtR may be a better predictor for risk of hypertension than BMI. Finally, parameter estimations which are insignificant with respect to statistics are usually removed in logistic regression analysis, whereas they can still be partly identified by comparison of spatial pattern before and after adjustment of corresponding predictors in the SCM analysis.

It is worth remarking that age was a risk factor for hypertension for both genders in the multiple logistic regression analysis, whereas the SCM found that the risk of hypertension decreased slightly for males 60 years of age or older compared with the middle-aged group. This may be due in part to the effect of age being substituted by percentages of elderly people in each region. Therefore, the logistic regression analysis reflected effects of variables at the individual level, but the SCM reflected the mean effect at the regional level by utilizing variables that may be more stable.

Results of the SCM pointed out that the mechanism of age on hypertension may be different between males and females, which could be related to their physiological, psychological and lifestyle characteristics, such as: (1) menopause in females may cause physiological changes [53] (i.e., obesity resulting from changes in hormone levels), thereby increasing the risk of hypertension, which we found in those with 60–74 years of age; and (2) the decreased risk of hypertension for males after retirement to some extent may be due to a decline in work and life pressure, the reduction of smoking or alcohol consumption for socializing and therefore more concern about their health. In addition, the SCM showed that there may be other shared risk factors for hypertension in addition to WHtR > 0.5 at the regional level.

Finally, the SPR for hypertension in the middle-aged (45–59 years of age) and elderly (60+ years of age) in ZN was larger than that of ZS, whereas the SCM suggested spatial pattern of coastal to inland for females, but no longer obvious spatial patterns as previously found for males. In fact, the SPRs are vulnerable to extreme values due to the instability of ratio, which may result in a misleading visual effect on map. However, the SCM modeling was able to “borrow strength” from adjacent areas and dependent diseases which rendered a more robust disease rate.

The SCM leads to a more robust estimate of parameters, which in turn, may improve the understanding of the etiology of disease that may guide disease interventions in a cost effective way [33]. What’s more, the SCM procedure may effectively analyze data containing missing values. However, caution should be taken, as the accuracy of spatial analysis is partly dependent on the scale of available dataset. Thus, it may be necessary to conduct analysis at finer scale, such as at the county level to improve the precision of estimation in future research.

Several limitations of this study merit discussion. One limitation of the current study is that hypertension is self-reported (abbreviated as HP) and this may be inaccurate and a potential cause of bias in epidemiological studies. Ideally, hypertension is defined by the current anthropometric measurements as systolic blood pressure ≥ 140 or diastolic pressure ≥ 90 mm Hg or current use of antihypertensive medication (abbreviated as bioHP). However, there is evidence that self-reported hypertension is valid [24]. Furthermore, our data show that there are as many as 92.4 % (387/419) participants diagnosed with both HP and bioHP. And the average time gap between his or her age in our study period and age of being diagnosed with hypertension is 3 years, with two extreme values (e.g. 10 years), for those diagnosed with HP while not with bioHP (32/1267, 2.5 %). Therefore, we believe that the impact of false-positive HP cases on the manuscript will be minor. The other limitation is that primary hypertension cases and secondary hypertension cases are not discriminated and this may also be a potential cause of bias in epidemiological studies. However, primary hypertension is much more prevalent than secondary hypertension. Thus, the impact of secondary hypertension cases on the results of the manuscript will be limited.

## Conclusion

Through the use of the SCM, our study identified risk factors for hypertension for the middle-aged and elderly population in Zhejiang Province, China at the regional level. Our SCM analysis determined that the spatial pattern of hypertension risk for the middle-aged and elderly populations in Zhejiang Province in eastern China is quite different for males and females. Furthermore, WHtR continues to be a simple and effective predictor of hypertension risk for males at the regional level.

The SCM modeling used in our study took advantage of the flexibility of Bayesian hierarchical modeling and the easy implementation of the MCMC technique while taking into account the dependency not only among diseases but also among spatially varying variables. Thus, the posterior estimates of the RR is more stable (e.g., the extreme effect of expected counts in Jiaxing, ZN for males was filtered out by spatially smoothing), therefore the problem of instability in the SPR analysis can be effectively avoided by the SCM.

We believe our study contributes to the medical literature due to: (1) our focusing on a non-rare disease (e.g., hypertension) that enable us to broaden the existing SCM by pinpointing fixed effects according to disease or gender specific regional covariates; thus determining that the SCM can be used to quantify disease or gender specific fixed effects and be applied to non-rare diseases for identifying risk factors at the regional level; (2) the extensions of the modeling procedure of SCM to assume missing covariates are normally distributed verifies that the SCM procedure may effectively analyze data containing missing values; and (3) comparing the performance of the SCM model with a more traditional logistic regression model also substantiates the utility of the SCM. If possible, future research should be conducted using the SCM at a finer scale (i.e., county) to identify potential risk factors for non-rare diseases.

## Abbreviations

bioHP, hypertension defined by the current anthropometric measurements as systolic blood pressure ≥ 140 or diastolic pressure ≥ 90 mm Hg or current use of antihypertensive medication; BMI, body mass index; CHARLS, China Health and Retirement Longitudinal Study; CI, confidence interval; DIC, deviance information criterion; HP, self-reported hypertension; HRS, Health and Retirement Study; MCMC, Markov chain Monte Carlo; MLE: maximum likelihood estimator; OR, odds ratios; RR, relative risk; SAGE, Study on Global Ageing and Adult Health; SCM, Shared Component Model; SPR, standardized prevalence ratio; WHO, World Health Organization; WHtR, Waist -to-Height Ratio; ZN, northeast Zhejiang; ZS, southwest Zhejiang

## Additional files

Additional file 1: Data for logistic analysis. data.dta. It is the dataset supporting the conclusions of logistic analysis, which were performed in Stata (version 11, Stata Corp, College Station, USA). (DTA 145 kb) Additional file 2: Data for SCM analysis. data for SCM analysis.txt. It is the dataset supporting the conclusions of SCM analysis, which were run in the free software WinBUGS version 1.4.3 with the GEOBUGS version 1.2 add on. (TXT 880 bytes) Additional file 3: Figure S2. Posterior mean of RR adjusted for obese for Zhejiang Province in 2012. (a), (b) denotes posterior mean of RR adjusted for obese for Zhejiang Province in 2012, by gender. (DOCX 266 kb) Additional file 4: Figure S3. Autocorrelation and convergence of RR for WHtR, male. Autocorrelation and convergence of RR for WHtR, male. (DOCX 19 kb) Additional file 5: Figure S5. Kernel density of RR for WHtR for females. (a) and (b), Kernel density of RR for WHtR for females. The sampling value and frequency of RR for WHtR are expressed in horizontal and vertical axis respectively. Two parallel MCMC chains were run for each model with samples size of 10,000. (DOCX 87 kb)

## Footnotes

1 City is larger than county in China.

## References

[1] Liu L, Wu Z, Zhu D (2010). Guidelines for Prevention and Treatment of Hypertension in China (revised edition, 2010).

[2] Lloyd-Sherlock P, Beard J, Minicuci N (2014). Hypertension among older adults in low-and middle-income countries: Prevalence, awareness and control. Int J Epidemiol.

[3] Non-Communicable Chronic Disease Center for Disease Control and Prevention, Chinese Center for Disease Control and Prevention (2012). Report on chronic diseases and risk factor surveillance in China (2010).

[4] Liao H, Mei JI, Zhang K (1999). A study on risk factors of essential hypertension (EHT) among the rural middle-aged and elderly people. Chin J Prev Contr Chron Non-commun Dis.

[5] Wang J-s, Yu J-m, Hu D-y (2009). Prevalence of hypertension and risk factors among young adults aged 20 to 44 years in Beijing Community. Chin J Hypertens.

[6] Wang L-n, Li CAO, Jing-yi Z (2008). The investigation of risk factor s of hyper tension among adult residents in Hebei Province. Chin J Prev Contr Chron Non-commun Dis.

[7] Xin-jian L, Xu J-y, Hai-hong Y (2010). Case-control study on risk factors for hypertension among Shanghai residents. Chin J Prev Contr Chron Dis.

[8] Luo L, Rong-sheng L, Ping Y (2003). Meta-analysis of risk factor on hypertension in China. Chin J Epidemiol.

[9] Xiao J*, Yang M, Zong L (2012). Application of logistic regression and log-linear model on the study of risk factors of hypertension. Chin J Prev Contr Chron Dis.

[10] Hong-yan L, Hui P, Ao-bo L (2009). Risk factors of essential hypertension in China: A meta-analysis. Chin J Cardiovasc Med.

[11] Niskanen L, Laaksonen DE, Nyyssönen K (2004). Inflammation, abdominal obesity, and smoking as predictors of hypertension. Hypertension.

[12] Ishikawa K, Ohta T, Zhang J (1999). Influence of age and gender on exercise training-induced blood pressure reduction in systemic hypertension. Am J Cardiol.

[13] Helmers KF, Baker B, O’Kelly B (2000). Anger expression, gender, and ambulatory blood pressure in mild, unmedicated adults with hypertension. Ann Behav Med.

[14] Momtaz YA, Hamid TA, Yusoff S (2012). Loneliness as a risk factor for hypertension in later life. J Aging Health.

[15] Lopez Garcia E, Faubel R, GuallarCastillon P (2009). Self-reported sleep duration and hypertension in older Spanish adults. J Am Geriatr Soc.

[16] Kagan A, Faibel H, Ben-Arie G (2007). Gender differences in ambulatory blood pressure monitoring profile in obese, overweight and normal subjects. J Hum Hypertens.

[17] Pechère-Bertschi A, Burnier M (2004). Females sex hormones, salt, and blood pressure regulation*. Am J Hypertens.

[18] Shunichi K, Kazuo M, Genjiro K (1992). A gender difference in the association between salt sensitivity and family history of hypertension. Am J Hypertens.

[19] Sesso HD, Cook NR, Buring JE (2008). Alcohol consumption and the risk of hypertension in women and men. Hypertension.

[20] Witteman J, Willett WC, Stampfer MJ (1990). Relation of moderate alcohol consumption and risk of systemic hypertension in women. Am J Cardiol.

[21] Cappuccio FP, Stranges S, Kandala N (2007). Gender-specific associations of short sleep duration with prevalent and incident hypertension the Whitehall II study. Hypertension.

[22] Hagberg JM, Park J, Brown MD (2000). The role of exercise training in the treatment of hypertension. Sports Med.

[23] Hui L (2009). Gender Differences of Prevalence and Control of Hypertension in Rural Shandong, China.

[24] Kaplan MS, Huguet N, Feeny DH (2010). Self-reported prevalence of hypertension and income among older adults in Canada and the United States. Soc Sci Med.

[25] de Gaudemaris R, Lang T, Chatellier G (2002). Socioeconomic inequalities in prevalence of hypertension and care the IHPAF study. Hypertension.

[26] Gangwisch JE, Heymsfield SB, Boden-Albala B (2006). Short sleep duration as a risk factor for hypertension analyses of the first national health and nutrition examination survey. Hypertension.

[27] Ma Y-x, Bing Z, Wang H-j (2011). The Effect of Alcohol Consumption on Prevalence of Hypertension among Adults Residents from 9 Provinces of China. Chin J Prev Contr Chron Dis.

[28] Wu X-g, Xiu-fang D, Guang-yong H (2003). Prevalence and relevant influence factors of isolated systolic hypertension in the elderly in China. Chin J Cardiol.

[29] Jie C, Xiu-li Z, Wu F (2005). Epidemiology of obesity and overweight and relation thereof to the prevalence of hypertension in 14 provinces/municipality in China. Natl Med J China.

[30] Bowman TS, Gaziano JM, Buring JE (2007). A prospective study of cigarette smoking and risk of incident hypertension in women. J Am Coll Cardiol.

[31] Knorr Held L, Best NG (2001). A shared component model for detecting joint and selective clustering of two diseases. J R Stat Soc Ser A (Stat Soc).

[32] Held L, Natário I, Fenton SE (2005). Towards joint disease mapping. Stat Methods Med Res.

[33] Schur N, Gosoniu L, Raso G (2011). Modelling the geographical distribution of co-infection risk from single-disease surveys. Stat Med.

[34] Held L, Graziano G, Frank C (2006). Joint spatial analysis of gastrointestinal infectious diseases. Stat Methods Med Res.

[35] Downing A, Forman D, Gilthorpe MS (2008). Joint disease mapping using six cancers in the Yorkshire region of England. Int J Health Geogr.

[36] Kazembe LN, Muula AS, Simoonga C (2009). Joint spatial modelling of common morbidities of childhood fever and diarrhoea in Malawi. Health Place.

[37] Earnest A, Beard JR, Morgan G (2010). Small area estimation of sparse disease counts using shared component models-application to birth defect registry data in New South Wales. Australia Health Place.

[38] Ibáñez-Beroiz B, Librero-López J, Peiró-Moreno S (2011). Shared component modelling as an alternative to assess geographical variations in medical practice: Gender inequalities in hospital admissions for chronic diseases. BMC Med Res Methodol.

[39] Strong M, Pearson T, Macnab YC (2012). Mapping gender variation in the spatial pattern of alcohol-related mortality: A Bayesian analysis using data from South Yorkshire, United Kingdom. Spat Spatiotemporal Epidemiol.

[40] Yaohui Zhao, Strauss J, GonghuanYang, et al. China Health and Retirement Longitudinal Study (CHARLS). 2012. http://charls.ccer.edu.cn/zh-CN. 10.1093/ije/dys203 PMC3937970 23243115

[41] Committee on Aging. Zhejiang Elderly Population and an Aging Career Statistical Bulletin (2014); 2015.

[42] Truelsen T, Begg S, Mathers C (2000). The global burden of cerebrovascular Disease (Global Burden of Disease 2000).

[43] Yaohui Zhao, John Strauss, Gonghuan Yang, etc. CHINA HEALTH AND RETIREMENT LONGITUDINAL STUDY—2011-2012 NATIONAL BASELINE USERS’ GUIDE. In: Appendix D. Detailed description of biomarkers. 2013. p.41-46. http://charls.ccer.edu.cn/en/page/data/2011-charls-wave1. Accessed Feb 2013, Updated April 2013.

[44] Coorperative Meta-analysis Group of China Obesity Task Force (2002). Predictive values of body mass index and waist circumference to risk factors of related disease in Chinese adult population. Chin J Epidemiol.

[45] Ware LJ, Rennie KL, Kruger HS (2014). Evaluation of waist-to-height ratio to predict 5 year cardiometabolic risk in sub-Saharan African adults. Nutr Metab Cardiovasc Dis.

[46] Li W, Chen I, Chang Y (2013). Waist-to-height ratio, waist circumference, and body mass index as indices of cardiometabolic risk among 36,642 Taiwanese adults. Eur J Nutr.

[47] Besag J, York J, Mollié A (1991). Bayesian image restoration, with two applications in spatial statistics. Ann Inst Stat Math.

[48] Mollié A (1996). Bayesian mapping of disease. Markov chain Monte Carlo in practice.

[49] Brooks SP, Gelman A (1998). General methods for monitoring convergence of iterative simulations. J Comput Graph Stat.

[50] Spiegelhalter DJ, Best NG, Carlin BP (2002). Bayesian measures of model complexity and fit. J R Stat Soc Series B (Stat Methodol).

[51] Onicescu G, Hill EG, Lawson AB (2010). Joint disease mapping of cervical and maleoropharyngeal cancer incidence in blacks and whites in South Carolina. Spat Spatiotemporal Epidemiol.

[52] Xiao -zhen L, Huang Y-b, Zhan S-y (2009). The predictive value of waist -height ratio to discriminate adult hypertension: A Meta-analysis. Chin J Prev Contr Chron Dis.

[53] Luoto R, Sharrett AR, Schreiner P (2000). Blood pressure and menopausal transition: the Atherosclerosis Risk in Communities study (1987-95). J Hypertens.

