# Supplementary material for: Spatial analysis of gender variation in the prevalence of hypertension among the middle-aged and elderly population in Zhejiang Province, China
Source: BMC Public Health. 2016 May 26;16:447. doi: 10.1186/s12889-016-3121-y (PMC4882773; doi:10.1186/s12889-016-3121-y)
Supplement: Additional file 3: Figure S2. — Posterior mean of RR adjusted for obese for Zhejiang Province in 2012. (a), (b) denotes posterior mean of RR adjusted for obese for Zhejiang Province in 2012, by gender. (DOCX 266 kb) [file 12889_2016_3121_MOESM3_ESM.docx]

Additional file 3

| 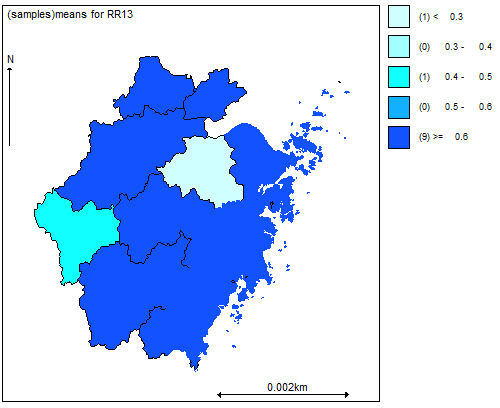  Figure S2 (a) RR adjusted for obese, males | 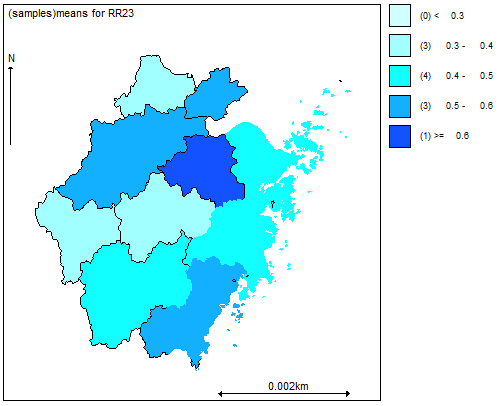 (b) RR adjusted for obese, females |
| --- | --- |

Figure S2 (a), (b) denotes posterior mean of RR adjusted for obese for Zhejiang Province in 2012, by gender
