# Supplementary material for: Spatial analysis of gender variation in the prevalence of hypertension among the middle-aged and elderly population in Zhejiang Province, China
Source: BMC Public Health. 2016 May 26;16:447. doi: 10.1186/s12889-016-3121-y (PMC4882773; doi:10.1186/s12889-016-3121-y)
Supplement: Additional file 4: Figure S3. — Autocorrelation and convergence of RR for WHtR, male. Autocorrelation and convergence of RR for WHtR, male. (DOCX 19 kb) [file 12889_2016_3121_MOESM4_ESM.docx]

Additional file 4

Figure S3 Autocorrelation and convergence of RR for WHtR, male
