# Supplementary material for: Spatial analysis of gender variation in the prevalence of hypertension among the middle-aged and elderly population in Zhejiang Province, China
Source: BMC Public Health. 2016 May 26;16:447. doi: 10.1186/s12889-016-3121-y (PMC4882773; doi:10.1186/s12889-016-3121-y)
Supplement: Additional file 5: Figure S5. — Kernel density of RR for WHtR for females. (a) and (b), Kernel density of RR for WHtR for females. The sampling value and frequency of RR for WHtR are expressed in horizontal and vertical axis respectively. Two parallel MCMC chains were run for each model with samples size of 10,000. (DOCX 87 kb) [file 12889_2016_3121_MOESM5_ESM.docx]

Additional file 5

Figure S5 (a) with Zhoushan (b) without Zhoushan

Figure S5 (a) and (b), Kernel density of RR for WHtR for females. The sampling value and frequency of RR for WHtR are expressed in horizontal and vertical axis respectively. Two parallel MCMC chains were run for each model with samples size of 10,000.
